# Joint Power and Channel Allocation for Non-Orthogonal Multiple Access in 5G Networks and Beyond

**DOI:** 10.3390/s23198040

**Published:** 2023-09-23

**Authors:** Qusay Alghazali, Husam Al-Amaireh, Tibor Cinkler

**Affiliations:** 1 Faculty of Electrical Engineering and Informatics, Budapest University of Technology and Economics (BME), 1117 Budapest, Hungary; husam.al-amaireh@edu.bme.hu (H.A.-A.); cinkler@tmit.bme.hu (T.C.); 2 Faculty of Electronics, Telecommunications and Informatics, Gdańsk University of Technology, G. Narutowicza 11/12, 80-233 Gdańsk, Poland; 3 ELKH-BME Cloud Applications Research Group, 1111 Budapest, Hungary

**Keywords:** 5G and beyond, resource allocation, non-orthogonal multiple access (NOMA), power allocation, fractional transmit power control (FTPC)

## Abstract

Spectral efficiency is a crucial metric in wireless communication systems, as it defines how much information can be transmitted over a given amount of spectrum resources. Non-orthogonal multiple access (NOMA) is a promising technology that has captured the interest of the wireless research community because of its capacity to enhance spectral efficiency. NOMA allows multiple users to share the same frequency band and time slot by assigning different power levels and modulation schemes to different users. Furthermore, channel assignment is a critical challenge in OFDMA-NOMA systems that must be addressed to achieve optimal performance. In this context, we propose a solution for both channel and power assignment based on channel condition by splitting the problem into two parts: first, we introduce a novel algorithm to solve the channel user allocation problem, which we refer to as Channel User Sorting and Filling (CUSF). Then, we solve the power allocation problem in two steps: we apply the water filling algorithm at the power assignment and then we implement the Fractional Transmit Power Control (FTPC) algorithm in the NOMA power assignment.

## 1. Introduction

Numerous 5G business models and applications are continuously emerging and developing due to the rapid growth of the Internet of Things (IoT), cloud services, and pervasive mobile devices and applications. To efficiently address the rising demand for bandwidth and services in this expanding environment, cutting-edge wireless technologies must be introduced [1].

The superiority of NOMA systems was theoretically shown in the straightforward scenario of a single base station and two users [2]. Since then, other works using various radio techniques and scenarios have addressed the assignment problem. The base station receives all signals in the up-link direction and can easily decode and cancel the individual data streams in a specific order. Each user must contend with this interference in the down-link direction, since each user receives both their signal and all of the signals meant for the other users assigned to the same channel. Therefore, the receiver attempts to eliminate the signals interfering with its signal reception whenever it is practical. This results in a set of restrictions on the minimum rate that the receiver observes for each data stream to be terminated, which makes the receiver’s job substantially more complex. The advantages of NOMA compared to traditional systems can be summarized as enhanced flexibility, more equitable access, and more significant spectral efficiency.

Since interference significantly impacts NOMA, channel and power optimization are crucial to its performance. Over the past 20 years, much research work has been carried out on power and sub-channel allocation [3]. The challenge of joint optimization of user association and power regulation to maximize the overall spectral efficiency is proposed, assuming that user-specific quality-of-service and total power transfer are guaranteed. The mixed-integer non-convex programming issue is addressed using a new transformation technique, which initially demonstrated that the non-convex channel allocation issue could be resolved nearly optimally in the Lagrangian dual domain [4].

In NOMA systems, attempting to overlay all users onto a single resource block is inefficient and unpractical in real systems and scenarios, due to the extensive decoding delay and the potential for severe error propagation in the Successive Interference Cancellation (SIC) process at the receiver. Hence, it becomes essential to decrease the count of users being superimposed on a given channel by distributing users considering the conditions of the channels and according to the complexity and delay requirements of the SICs. This process will result in groups of users, and each group can be handled as a NOMA group. By Employing effective algorithms for user grouping and power allocation, the signal interference can be mitigated, which leads to overall system capacity enhancement.

In this paper, we show that the joint resource and power allocation is a non-convex and NP-hard problem. Furthermore, we decouple the sub-channel and power allocation problems, We show that sub-channel allocation can be viewed as a matching procedure, where users and sub-channels are two sets that need to be paired together. This pairing aims to maximize the achievable data rate between them. We propose a novel resource allocation scheme by dividing the non-convex problem; first, we introduce a new one-to-many resource allocation by assuming equal power allocation over sub-channels. The main idea is to use one-to-many heuristics to predict the optimal resource allocation. Then, we tackle the power allocation by deriving an iterative water-filling scheme for sum-rate maximization for down-link NOMA systems. The optimal scheme can be obtained by formulating a power allocation as a sum rate maximization problem and then exhaustively searching for the solution to the formulated problem.

The rest of this paper is organized as follows. Related work is introduced in Section 2. The system model and problem formulation are introduced in Section 3. The proposed resource allocation is detailed in Section 4. The proposed power allocation scheme is presented in Section 5. The simulation results are shown in Section 6. Finally, the conclusion is drawn in Section 7.

## 2. Related Work

Numerous studies have gone into great detail concerning optimizing resource management for NOMA transmission. The subject has attracted a great deal of interest in the literature. The discipline of wireless communications optimization has made outstanding strides in recent years as wireless communications technology continues to develop and grow. One of the key developments in this area was published in [4], which showed that using the Lagrangian dual domain to solve the non-convex channel allocation problem nearly optimally as possible.Since then, numerous studies have concentrated on resolving allocation issues in various contexts and with different radio technologies.

For instance, Ref. [5] proposed cross-layer solutions, Ref. [6] investigated cognitive radio, Ref. [7] analyzed small cell and heterogeneous networks, the authors in [8] explored cloud radio access networks, and in [9], they investigated Multiple-Input Multiple-Output (MIMO) systems. These studies exemplify the extensive research efforts to optimize wireless communications systems through effective resource allocation strategies. By tackling the challenges posed by non-convex optimization problems, researchers have made significant progress toward developing efficient and practical optimization techniques for wireless networks. These efforts are essential for meeting the ever-increasing demands of modern society for reliable and high-speed wireless connectivity. In [10], a straightforward case study using one base station and two users illustrated the NOMA system’s theoretical superiority. The findings of this study provide compelling evidence that NOMA may be preferable to other multiple-access systems, even in critical situations. The authors in [11] studied different NOMA techniques, challenges, and its implementations in 5G and beyond networks.

As part of the extensive research on optimizing resource management for NOMA transmission, the work in [12] presents a proposed framework to address a resource management problem in two-user NOMA systems. The authors aim to enhance the sum capacity of the system by first providing the minimum Quality of Service (QoS) for one mobile user and then allocating the remaining power to the other user to maximize overall capacity. This approach provides a potential solution to the challenge of resource allocation in NOMA transmission, and contributes to the ongoing efforts to improve the performance of these systems. The key challenge in managing resource allocation for NOMA transmission is addressing the multi-user interference stemming from non-orthogonal channel access. However, this challenge is complicated by the non-convex essence of the allocation problem, which requires advanced and complex algorithms to solve. A potential solution to this challenge is presented in [10], where the authors demonstrate that the NOMA Full-Duplex (NOMA-FD) mode is theoretically feasible and can provide substantial gains over NOMA Half-Duplex (NOMA-HD) and orthogonal multiple access. However, this approach demands proper co-channel multi-user interference management for optimal implementation. The greedy asynchronous distributed interference avoidance algorithm (GADIA) based power allocation strategy for NOMA-based communications was discussed in [13].

The authors in [14] presented a price-based power optimization scheme for down-link wireless networks to maximize both revenues and the average achievable rate of the network. To achieve this objective, the authors adopted a game-theoretic approach. Since the resulting optimization problem was non-convex, they decoupled it into more manageable sub-problems and utilized an alternating optimization algorithm to obtain an efficient solution. By doing so, they were able to effectively address the complexity of the problem and provide a viable approach for optimizing power allocation in wireless networks.

Several power control algorithms have been proposed for NOMA systems, such as the algorithm proposed in [15], which aims to maximize the transmission rate of users while minimizing the transmission power. The authors in [15] proposed a distributed power adaptive algorithm that adjusts the transmission power of a user based on the signal quality of adjacent users. These algorithms can help improve the performance of NOMA systems by optimizing power allocation. A low-complexity power allocation method was put forth in [16] to enhance the weighted sum capacity in down-link NOMA systems. The method took into account both a two-user case and a multi-user one. It employed closed-form solutions to tackle the non-convex optimization issue effectively.

The effect of power distribution on the equity of the down-link NOMA system was examined in [17]. The authors of [18] suggested an energy-efficient power distribution plan that addressed the Single Carrier NOMA (SC-NOMA) system’s sum rate maximization problem. Energy-efficient power allocation for a hybrid system with NOMA connected to OMA was researched in [19]. For unsatisfactory NOMA-based down-link heterogeneous networks, the problem of cluster formation and power-bandwidth allocation is addressed in [20]. As a function of QoS requirements, SIC efficiency, and allotted bandwidth, references [21,22] concentrate on the up-link resource allocation and user pairing and determine the greatest practical NOMA cluster size.

In [23], the authors accomplished energy-efficient resource management in NOMA Heterogeneous Networks (HetNets) by employing a transformation technique that converted the original non-convex optimization problem into a convex problem. Subsequently, they employed a dual method for effective sub-channel and power allocation, enabling efficient utilization of network resources while maintaining energy efficiency. A plan to optimize user association and spectrum allocation was put out in reference [24]. The strategy is intended to boost system performance while considering the fairness restriction. However, NOMA networks are not covered by the present approaches, which exclusively deal with the problem of user association in conventional heterogeneous networks.

The performance of NOMA for massive MIMO (mMIMO) networks, which is dependent on beam-forming and user clustering, was studied by the authors in [25]. The work in [26] presents a distributed approach for resource allocation and interference management in wireless networks, focusing on energy efficiency. The proposed solution allows for flexible and dynamic resource partitioning between macro and small cells, enabling energy-saving resource allocation. In their study, the authors in [27] presented a straightforward NOMA system configuration involving a single base station and two users. The analysis considered the Nakagami-M fading channel model, accounting for the statistical characteristics of the channel state information. Specifically, the authors formulated the outage probability of the NOMA network, offering insights into the system’s performance under the influence of fading channels. While NOMA offers several advantages, such as high spectral efficiency and support for massive connectivity, its successful implementation is not without challenges. One significant challenge arises from the large number of users sharing the same system resources, which enormously increases the complexity of the SIC process at the receivers. To address this issue, the performance of NOMA-FD systems has been investigated under the presumptions of imperfect SIC and channel state information (CSI) errors in studies such as [28,29]. Efficient utilization of network resources through optimal resource allocation is necessary to overcome limitations and improve the performance of NOMA-based systems [30].

## 3. System Model

In this section, we assume a down-link Base Station (BS) serving a set of users denoted by K, where K={1,2,…,K}. The available Bandwidth (BW) is divided into N sub-channels, where N={1,2,…,N}, and each sub-channel with a BW b=B/N. Also, CSI is fully available at BS. The users are assigned, according to NOMA, to the sub-channels based on their CSI in a manner that each sub-channel serves a sub-group of users. The total power transmitted is denoted by $P_{T}$, and each sub-channel is assigned a power $P_{n}$, such that $0\leq P_{n}\leq P_{T}$ with n∈N. Also, users are assigned power $p_{nk}$ where n∈N and k∈K, such that $\sum_{k\in K}p_{nk}\leq P_{n}$. Let the channel between a user *k* and BS on sub-channel *n* be $h_{kn}$. The channel matrix H between user *k* and BS can be seen as $H_{kn}\in C^{LxM}$ with L,M being the number of received and transmitted antennas, respectively.

Without loss of generality, we assume L=1 (this can be seen as a single antenna user or a single link between BS and one received antenna). In a rich multi-path environment (as is usually the case in cellular systems) and benefiting from the central limit theorem [31], the channel vector can be modeled as complex Gaussian with $h_{kn}∼CN\left(\mu_{kn},R_{kn}\right)$, where $\mu \in C^{M}$ represents the line of sight propagation, and the covariance matrix $R_{kn}\in C^{MxM}$ represents the variable nature of the channel. This model is called Rayleigh fading in case $\mu_{kn}=0$, otherwise it is a Rician channel. The off-diagonal elements in $R_{kn}$ represent the spatial directivity. 3GPP has modeled the channel attenuation as [32]:

(1) $$\begin{array}{c} \beta =-128.1-37.6\log_{10}d \end{array}$$

where *d* is the separation in kilometers. Furthermore, the noise power can be represented as:

(2) $$\sigma^{2}\left(\mathrm{dBm}\right)=-174+10\log_{10}\left(b\right)+n_{f}$$

where *b* is in Hertz, and $n_{f}$ is the hardware noise figure in dB. The data rate depends on Signal to Noise Ratio (SNR) and, hence, by assuming that the transmitted signals from each antenna are independent and identically distributed (i.i.d.) with a total power $p_{kn}$, we obtain:

(3) $$\begin{array}{cc} {\mathrm{SNR}}_{kn} & =\frac{p_{kn}\mathrm{tr}\left(R_{kn}\right)}{M\sigma^{2}},\\ \; & =\frac{p_{kn}g_{kn}}{\sigma^{2}} \end{array}$$

where tr(.) is the trace of a matrix, and $g_{kn}=\frac{\mathrm{tr}(R_{kn})}{M}$ is the average channel gain. From (3), we can notice that the SNR for a single user with the optimal preprocessing (like matched filtering) transforms the Multiple-Input Single-Output (MISO) channel into an equivalent Single-Input Single-Output (SISO) channel.

### 3.1. NOMA System

A significant classification for multiple access systems based on orthogonality has surfaced. Various techniques such as time, frequency, coding, and space can be utilized to achieve the orthogonality of communication resources. When these resources, or their combination, achieve orthogonality, the communication schemes can be classified as Orthogonal Multiple Access (OMA). In contrast, NOMA is gaining popularity due to its potential to enhance spectral efficiency, user fairness, reliability, and to accommodate more users. Numerous options, including coding and power, can be used to implement NOMA. Coding achieves multiplexing through the code domain. The code domain shares time and frequency, much like Code-Division Multiple Access (CDMA). In contrast, user-specific spreading sequences that are either sparse or non-orthogonal cross-correlation sequences with low correlation coefficients are used by code-domain NOMA [33]. On the other hand, NOMA power-domain multiplexing is generally regarded as less complex than code-domain. In power-domain NOMA, fractions of power (that sum up to a total of *P*) are allocated to users, thereby increasing the rate of OMA. The receiver differentiates users based on channel strength, with stronger channel users using the SIC method and weaker channel users treating the other signals as noise and decoding the correct signal. It is worth noting that the user’s power fraction is not solely dependent on the user’s channel condition; it can be controlled to regulate the rate per user to make the system fair.

The number of users served by the NOMA system or, more specifically, SIC, should be limited to a certain threshold mainly for two reasons: reducing complexity and minimizing error propagation. If we denote the sub-group of NOMA users on sub-carrier $c_{n}$ as $S_{n}\subseteq K$ such that $1\leq |S_{n}|\leq S$, then we can express users of main group as $u_{k}\in K$, and users belong to same NOMA sub-group on $c_{n}$ as $u_{sn}\in S_{n}$.

Consider that the users $u_{sn}$ are arranged in descending order according to their gains, such that:

(4) $$\begin{array}{c} g_{1n}\geq g_{2n}\geq g_{3n}\geq \ldots \geq g_{Sn} \end{array}$$

Then, according to the NOMA principle, the power will be assigned as:

(5) $$\begin{array}{c} p_{1n}\leq p_{2n}\leq g_{3n}\leq \ldots \leq g_{Sn} \end{array}$$

The input signal $u_{jn}$ is received and subjected to SIC by subtracting the signal intended for a later receiver from the composite signal. This leads to an improvement in the signal-to-interference-plus-noise ratio (SINR). To decode its own signal, $u_{jn}$ first decodes the interfering signals intended for the later receivers $u_{in}$, where i>j and $i,j\in S_{n}$. The interfering signals with lower order are not decoded and are treated as noise. Thus, the SINR prior to SIC can be expressed as follows:

(6) $$\begin{array}{cc} {\mathrm{SINR}}_{jn} & =\frac{p_{jn}g_{jn}}{\sum_{i=1,i\neq j}^{S}p_{in}g_{jn}+\sigma_{n}^{2}}, \end{array}$$

Then, after SIC, the estimated SINR is written as:

(7) $$\begin{array}{c} {\mathrm{SINR}}_{jn}=\frac{p_{jn}g_{jn}}{\sum_{i=1}^{j-1}p_{in}g_{jn}+\sigma_{n}^{2}} \end{array}$$

Based on the SINR from (7), the user rate can be expressed as:

(8) $$\begin{array}{c} R_{jn}=\log_{2}\left(1+\frac{p_{jn}g_{jn}}{\sum_{i=1}^{j-1}p_{in}g_{jn}+\sigma_{n}^{2}}\right) \end{array}$$

### 3.2. Joint Channel and Power Allocation

In our work, we consider the system performance as the total sum rate of all users. Then, the optimization problem is written as:

(9a) $$\begin{array}{cc} P1:\max_{x,p} & \sum_{k\in K}\alpha_{k}\sum_{n\in N}R_{kn}x_{kn} \end{array}$$

(9b) $$\begin{array}{cc} s.t. & \sum_{k\in K}\sum_{n\in N}p_{kn}\leq P_{T}, \end{array}$$

(9c) $$\begin{array}{cc} \; & \sum_{n\in N}p_{kn}\leq P_{n} \end{array}$$

(9d) $$\begin{array}{cc} \; & s_{l}\leq \sum_{k\in K}x_{kn}\leq S \end{array}$$

(9e) $$\begin{array}{cc} \; & x_{kn}\in \left\{0,1\right\} \end{array}$$

(9f) $$\begin{array}{cc} \; & 0\leq p_{kn} \end{array}$$

The objective is to maximize the weighted utility function (9a), where $R_{kn}$ can be found in (8). $\alpha_{k}$ is the weight coefficient of $u_{k},k\in K$. The choice of these weights significantly impacts how resources are distributed among the users, and these weights can be employed to guide the resource distribution towards different objectives, such as giving priority to specific users and ensuring fairness by assigning a higher weight to a user with a relatively weaker channel. Constraint (9b) guarantees that the power budget will not be exceeded. Constraint (9c) is to ensure that the total sub-channel allocated power does not exceed a certain threshold. Constraints (9d) and (9e) ensure, respectively, that each sub-channel is both upper and lower bounded by the number of users, and $u_{k}$ is multiplexed in $c_{n}$. Constraint (9f) ensures that power gives positive values.

The problem P1 is non-convex due to the existence of the binary object and interference term in the objective function. Furthermore, it is NP-hard; this can be seen if we make S=1, which reduces into Orthogonal Frequency Division Multiple Access (OFDMA), where its NP-hardness can be seen in [34]. Because of the NP-hardness of this problem, we can no longer insist on having an efficient algorithm that can find its global optimum in polynomial time. Instead, we have to settle for less ambitious goals and find approximate solutions. In the next section, we address the sub-channel and power allocation problems independently.

## 4. One-to-Many NOMA Algorithm

In this algorithm, we are looking for matching between sub-channels and users without considering power assignment. We assume that channels have preferences over users in a way that they are able to rank order them based on their channel gain, and the same goes for users. However, each channel will be able to choose more than one user, and users will be able to choose one channel in a one-to-many scenario.

As mentioned above, we consider two finite and disjoint sets K,N for users and channels, respectively. Each channel has preferences over users, and each user has preferences over channels. In our model, the preferences are considered transitive such that, if a user prefers channel $n_{x}$ over $n_{y}$, and prefers $n_{y}$ over $n_{z}$, then it definitely prefers $n_{x}$ over $n_{z}$. The preferences (ordered from best to worst) for both users and channels can be expressed as follows:

(10) $$\begin{array}{cc} F(u_{x}) & =n_{a},n_{b},u_{x},n_{c},\ldots \end{array}$$

(11) $$\begin{array}{cc} F(n_{x}) & =u_{a},u_{b},u_{c},n_{x},u_{d},\ldots \end{array}$$

It can be noticed from (10) that $u_{x}$ finds both $n_{a},n_{b}$ acceptable, and discards other channels below certain channel gain threshold. Also, the same applies for (11) in which channel $n_{x}$ accepts only users $u_{a},u_{b},u_{c}$ and discards $u_{d}$ because of channel gain. We also denote $n_{x}\geq_{u_{x}}n_{y}$ as: user $u_{x}$ prefers $n_{x}$ at least as $n_{y}$, and $u_{x}\geq_{n_{x}}u_{y}$ as: channel $n_{x}$ prefers $u_{x}$ at least as $u_{y}$. The expected outcome of such a model is that each user is matched to at most one channel, each channel is matched to a specific quota (based on NOMA complexity and error propagation requirements), and the matching is bilateral in a way that a user is paired with a channel if and only if the channel is paired with the user. Based on the above, let us define the matching process γ as follows:

1. |γ(u)|=1 for every user and γ(u)=u if γ(u)∉N;
2. |γ(n)|=S for every channel, any unfilled position with users will be filled with *n*;
3. |γ(u)|=n⇔u∈γ(n).

Since channels in the matching algorithms serve a specific quota of users, the matching algorithm should allow the channel to compare groups of users and compare different matching.

Next, inspired by the National Intern Matching Program (NIMP) [35], we present our modified algorithm, which we name the Channel User Sorting and Filling (CUSF) algorithm. The functioning of the algorithm is outlined as follows:

- Entry stage:
  First, the base station orders the users who have applied based on each channel’s ranking. Any user with an unacceptable channel gain is eliminated, and each user ranks the channels, and any channel with an unacceptable channel gain is eliminated from the users’ lists. Next, the lists enter a list processing, beginning with the matching stage.
- Matching stage:
  In the matching stage, the algorithm searches for user–channel pairs that are top-ranked in each other’s ranking. If no matches are found, it proceeds to the next step, where the second-ranked channel on each user’s ranking is compared with the top-ranked user in that channel’s ranking. The generic step seeks to find user–channel pairs such that the user is top-ranked on the channel’s ranking and the channel is ranked $k_{\mathrm{th}}$ by the user. If matches are found, the algorithm goes to the next stage.
- Provisional assignment and update stage:
  In this stage, k:1 matches are tentatively made, and each user who is the top-ranked choice of their $k_{\mathrm{th}}$ choice channel is tentatively assigned to that channel. The rankings of users and channels are then updated, and the algorithm returns to the start of the matching stage, which examines the updated ranking for new matches. The algorithm continues until no new tentative matches are found, at which point tentative matches become final.

The CUSF algorithm is shown in Algorithm 1.
**Algorithm 1** CUSF algorithmRank order both channel and user lists according to channels gains, respectively, as ζ,η**while** (there are requested S:1 matches)
Assign all items that have been marked as tentative;Remove any channels with lower ranks from the list of users who have been assigned;Eliminate any users who were tentatively assigned from the list of channels they ranked lower than their tentative assignment.**End**

### Stability Assumption: The Matching γ Is Stable If It Is Not Blocked by Any Channel-User Pair

A matching γ is said to be blocked by a channel n and a user u if they are currently not matched to each other in γ, but both would prefer to be matched with each other rather than their current assigned matches, where we consider (n,u) as a blocking pair because of the simultaneous occurrence of γ(u)≠n, $n{>}_{u}\gamma \left(u\right)$, and $u{>}_{n}\gamma \left(n\right)$.

**Theorem 1.**  *CUSF is a stable algorithm.*

**Proof.**  After the algorithm stops, each channel $n_{i}$ is paired with the best S options from its updated rank-ordered list. This is because the algorithm only stops when it is no longer possible to find tentative k:1 matches. The resulting matching is considered stable, as any user $u_{j}$ that was initially ranked higher by channel $n_{i}$ than one of its final choices has been removed from $n_{i}$ list due to being provisionally assigned to a higher-ranked channel on $u_{j}$ list. Therefore, the final assignment provides $u_{j}$ with a position that he prefers over $n_{i}$. Consequently, the final matching is not unstable in terms of any $n_{i}$ or $u_{j}$ of this kind.    □

At this stage, we should have a new channel-user assignment. Next, we move to power assignment-based CUSF algorithm outcome.

## 5. Power Allocation

After assigning channels to users, we look now into the power allocation. For this purpose, we divide the problem into two parts: allocating power per sub-carrier, then allocating power to users that are superimposed on a single carrier.

To allocate power per sub-channel, we consider sub-channels with only one user per sub-channel. Next, we establish an optimization problem based on maximizing the total sum rate as follows:

(12a) $$\begin{array}{cc} P2:\max_{p_{n}} & \sum_{n\in N}R_{n} \end{array}$$

(12b) $$\begin{array}{cc} s.t. & \sum_{n\in N}p_{n}\leq P_{T}, \end{array}$$

(12c) $$\begin{array}{cc} \; & 0\leq p_{n} \end{array}$$

where problem P2 is strictly convex with respect to $p_{n}$, and so it has a unique solution. We can solve P2 by Karush–Kuhn–Tucker (KKT) conditions. To simplify the notations, we assume that $\frac{g_{n}}{\sigma_{n}^{2}}=H_{n}$. Let us establish the Lagrangian function as follows:

(13) $$\begin{array}{c} L\left(p,\lambda \right)=\sum_{n\in N}log_{2}\left(1+p_{n}H_{n}\right)-\lambda \left(\sum_{n\in N}p_{n}-P_{T}\right) \end{array}$$

When we solve for $p_{n}$, and λ (see Appendix A for derivations), then (13) gives:

(14) $$\begin{array}{cc} p_{n} & =\frac{1}{\ln_{2}\lambda }-\frac{1}{H_{n}}, \end{array}$$

The power allocation in (14) is called water filling. From (13) and (14), we derive the procedure to obtain the optimal values for $p_{n}$ as follows:

(15) $$\begin{array}{c} \lambda_{i+1}=\lambda_{i}-A{\left[P_{T}-\sum_{n\in N}p_{n}\right]}^{+} \end{array}$$

where A is step size, and ${\left[.\right]}^{+}$ is a non-negative number. So far, the results give us power allocations considering only one user per channel. The next step is to allocate power for each sub-channel superimposed NOMA users. For this purpose, we benefit from Fractional Transmit Power Control (FTPC) [36] as follows:

(16) $$\begin{array}{c} p_{kn}=\frac{p_{n}}{\sum_{i\in S}H_{in}^{-\alpha_{\mathrm{FTPC}}}}H_{kn}^{-\alpha_{\mathrm{FTPC}}} \end{array}$$

where *S* number of superimposed users in sub-channel *n*, $0\geq \alpha_{\mathrm{FTPC}}\geq 1$ is the decay factor. The case of $\alpha_{\mathrm{FTPC}}=0$ corresponds to equal transmit power allocation among the users. The more $\alpha_{\mathrm{FTPC}}$ is increased, the more power is allocated to the user with lower channel gain $H_{kn}$. The power assignment algorithm is shown in Algorithm 2.
**Algorithm 2** Power allocationInitialize $\lambda_{\left(0\right)}>0$, set iteration number i=0, set differential tolerance value ξ**While** $|\lambda_{i+1}-\lambda_{i}|>\xi$ **do**Calculate $p_{n}$ from (14)Update $\lambda_{i+1}$ in (15)i←i+1**End while**.Calculate $p_{kn}$ from (16)

## 6. Results and Discussion

In the simulation section, we study the capacity of the system with the parameters mentioned in Table 1. Furthermore, we compare the scheme with another algorithm which is the Channel State Sorting-Pairing Algorithm (CSS-PA) [37]. The use of SIC in wireless communication requires a significant difference in signal-to-SINR between paired users to prevent error propagation, and CSS-PA addresses this by pairing a user with a good channel condition with one who has a bad channel condition. This improves fairness and increases the capacity of the system. Additionally, OFDMA is used to evaluate the impact of NOMA on the overall system performance.

**Figure 1:**
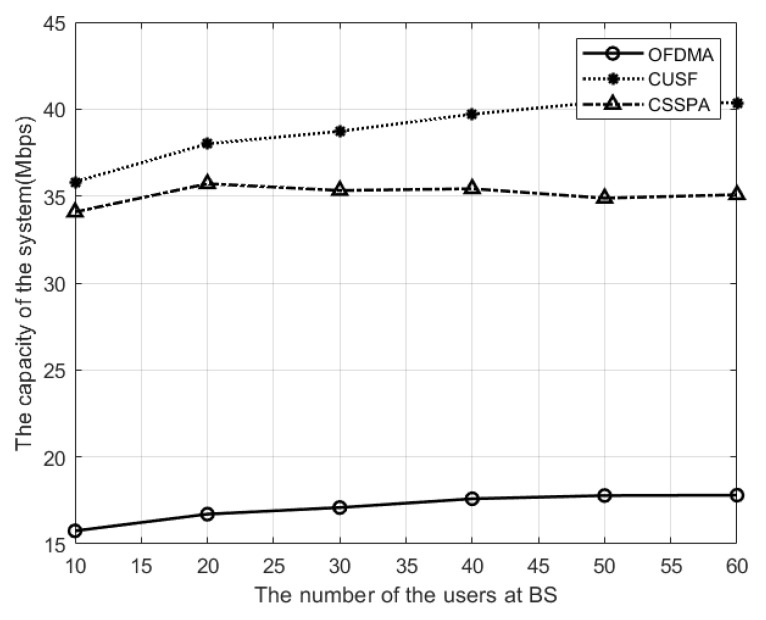
Capacity of the system versus different numbers of users.

Figure 1 illustrates the network capacity curve as the number of users in a cell increases from 10 to 60. The channel capacity is calculated by multiplying (8) with the bandwidth of each sub-channel, then we summed the capacities of all sub-channels. We assumed the bandwidth of each sub-channel equals the total bandwidth divided by the number of sub-channels. The capacity of the cell system also increases with the number of users.

CUSF provides the highest system capacity for the NOMA system compared to other algorithms studied. At 40 users, the CUSF algorithm outperforms CSS-PA by approximately 15% and OFDMA by 60%. This is due to the limitations of OFDMA for using only one user per sub-channel, which results in the BS being unable to fully utilize the spectrum resources.

**Figure 2:**
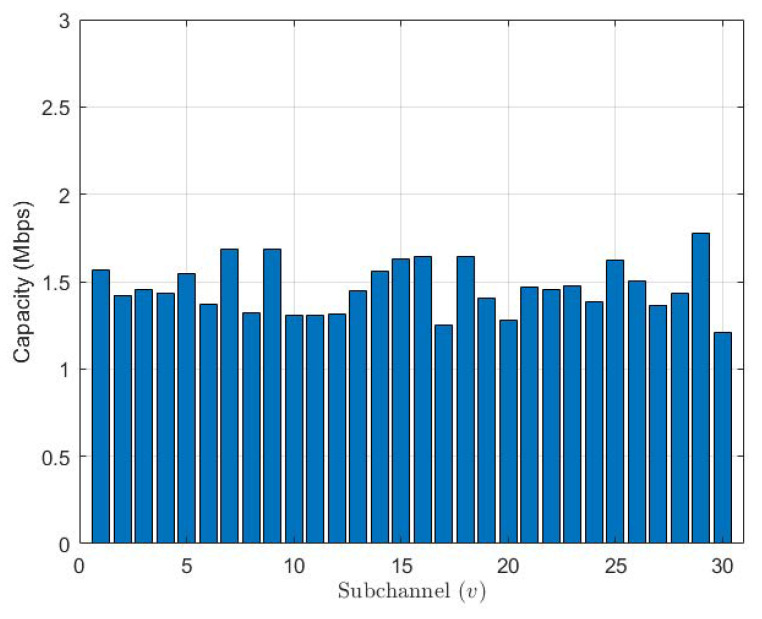
System capacity distributed over channels.

**Figure 3:**
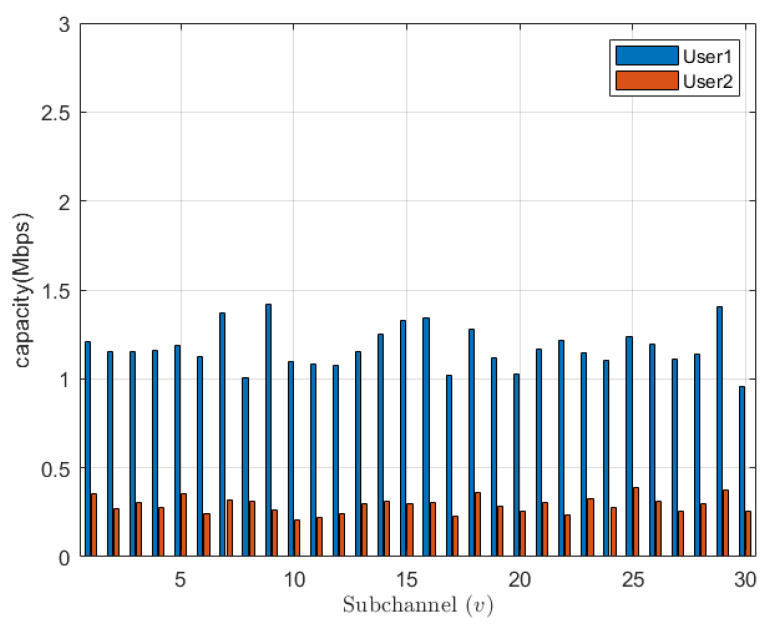
Capacity per user for per sub-channel.

In Figure 2, we show the capacity of the system distributed over channels. By studying the figure, despite different channel conditions (we have assumed a fading Rayleigh channel condition), we can notice that the algorithm manages to provide good fairness of capacity between channels. In other words, the system has distributed the users per channel in an optimized way based on their channel conditions. Furthermore, a detailed capacity distribution per user per sub-channel is shown in Figure 3. In the figure, we can see that the algorithm has optimally distributed the users on sub-channels in a way that each sub-channel serves a user with a good channel condition and a user with a severe channel condition, and this scenario will maximize the capacity per sub-channel and hence will increase the overall system capacity.

**Figure 4:**
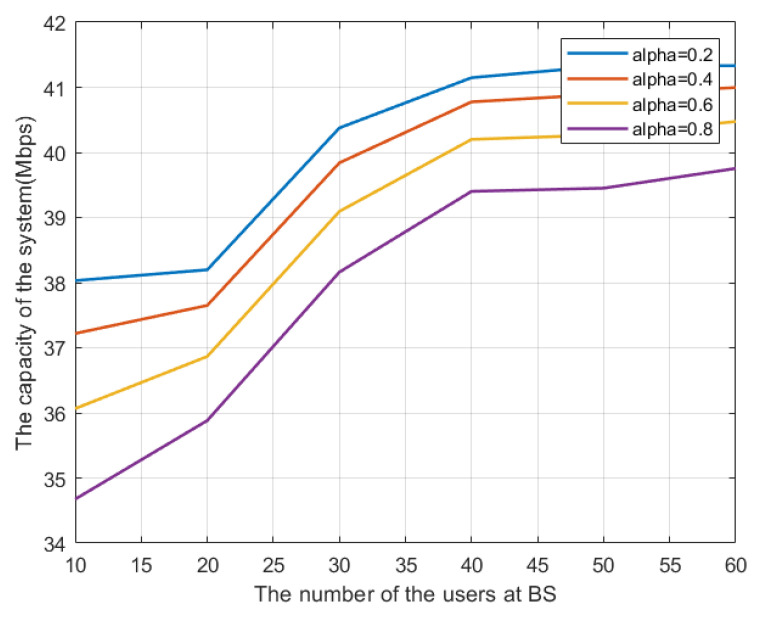
System capacity for different alpha values.

The impact of different power assignments is shown in Figure 4. It can be seen that the capacity increases inversely proportional to alpha. Furthermore, for the case of five channels and 10 users, we show a list of users sorted by each channel according to their respective gains before applying the CUSF algorithm in Table 2. Then, after applying the CUSF algorithm (before power assignment), we see the channel-user assignment in Table 3. According to the distribution of users, we see that the algorithm has, to a good degree, fairly distributed the users on channels, which helps in optimizing the overall system performance.

## 7. Conclusions

In this paper, we have introduced an optimization problem for channel user allocation. We have shown that the optimum channel allocation is non-convex and NP-hard. To solve this problem we have decoupled resource and power allocation into two parts. We have introduced a novel algorithm in which the channel user allocation is based on one-to-many channel allocation. Furthermore, we have introduced a power optimization problem, which we have solved in two steps: firstly, by introducing a convex problem for channel assignment, and secondly, by benefiting from the FTPC algorithm for NOMA power assignment. In future work, we will build upon the results and study the effect of applying CUSF on other multiple-access baselines, like conventional Multi-User Linear Precoding (MU–LP) [38]. 

## Figures and Tables

**Table 1:** The list of simulation parameters.

Simulation Parameters	Parameter Value
Cell radius	500 m
The minimum distance between BS and UEs	50 m
The minimum distance between user and user	40 m
System bandwidth	5 MHz
The maximum number of UTs	60
Noise power spectral density	−174 dBm/Hz
Difference tolerance in Algorithm 2	0.01
The base station peak power $P_{BS}$	30 dBm

**Table 2:** Channel list of users sorted according to their gain.

Channel Number	User Number									
1	3	1	2	5	6	8	4	7	10	9
2	3	4	1	6	10	2	8	5	7	9
3	1	4	3	5	2	8	6	9	10	7
4	3	1	5	9	2	4	10	8	6	7
5	1	3	4	2	8	9	10	5	7	6

**Table 3:** Channel user assignment using CUSF.

Sorted Users	Channel Assigned
1	3
3	1
4	3
2	1
5	4
9	4
6	2
8	5
10	2
7	5

## Data Availability

Not applicable.

## Appendix A. Power Allocation Water Filling Algorithm

The power allocation problem using the water-filling algorithm can be solved using the Karush–Kuhn–Tucker (KKT) conditions, which are a set of necessary conditions for a solution to be optimal for the optimization problem P2. The Lagrangian function for this problem is:

(A1) $$\begin{array}{c} L\left(p,\lambda \right)=\sum_{n\in N}log_{2}\left(1+p_{n}H_{n}\right)-\lambda \left(\sum_{n\in N}p_{n}-P_{T}\right) \end{array}$$

where λ is the Lagrange multiplier. The KKT conditions for this problem are:

1. Stationarity:
  (A2) $$\begin{array}{c} \frac{\partial L}{\partial p_{n}}=0,\mathrm{for}\;\;n=0,1,2,\ldots ,N \end{array}$$
2. Primal feasibility:
  (A3) $$\begin{array}{cc} \sum_{n=1}^{N}p_{n} & \leq P_{T} \end{array}$$
  (A4) $$\begin{array}{cc} p_{n} & \geq 0 \end{array}$$
3. Dual feasibility:
  (A5) $$\begin{array}{cc} \lambda & \geq 0 \end{array}$$

By obtaining the derivative of the Lagrangian with respect to $p_{n}$ of (A1) and the primal feasibility condition, we find the solution from the stationarity condition, which is written as:

(A6) $$\begin{array}{c} \frac{H_{n}}{\ln_{2}\left(1+H_{n}p_{n}\right)}=\lambda \end{array}$$

Then, we can use (A6) to solve for $p_{n}$, as:

(A7) $$\begin{array}{c} p_{n}=\frac{1}{\ln_{2}\lambda }-\frac{1}{H_{n}} \end{array}$$
